# International Text-Book of Surgery

**Published:** 1903-09

**Authors:** 


**International Text-book of Surgery.** By American and British Authors. Edited by J. COLLINS WARREN, M.D., and A. PEARCE GOULD. Vol. II.—*Regional Surgery*. Pp. 1072. Philadelphia: W. B. Saunders & Co. 1900.

We have already reviewed the first volume of this work (p. 61), and find that the general excellence and completeness are maintained in this second volume. The illustrations, many of them photographic reproductions, are excellent, and number nearly 500. Over thirty different authors have contributed, the greater number being American. The numerous contributors in special subjects have furnished a wealth of detail in the special branches, sometimes overlapping one another; but their special knowledge tends to exceed the possibilities of acquisition by the average student, for whom the book, as a whole, seems scarcely fitted. To him multiplicity of authorship is not necessarily an advantage, and the international character of the work involves the recognition of orthography different from our own, *e.g.* "caliber," "fiber," "goiter," a reference to authorities unfamiliar to the British student, and the allocation of different values to certain surgical procedures. It is thus curious to read in the section on the tongue that, as it were under compunction, "the Whitehead operation . . . must be described," the preference being given to a method of Kocher which the originator seems now to have abandoned. But it is perhaps more surprising that a British contributor should say that "prostatectomy should always be performed, to start with, by the suprapubic route," for the perineal route is very largely advocated in America. Or again, that suture to the periosteum of the twelfth rib, Tuffier's suggestion, should be the only method given for nephrorrhaphy. We think the description of pharyngeal adenoids and their treatment might be much improved upon. To surgeons the volume will afford much food for thought, especially the chapters by our American friends, whose practice and environment are somewhat different from our own. Such facts as that enlarged prostate scarcely ever occurs in the negro, and that noses are capable of such surgical improvement as depicted on pp. 85 to 89, are at least entertaining. The short nose may be made long to diminish the celestial snub, the large nose may be made small "to relieve melancholia," the aquiline becomes quite inviting after removal of the "bony hump," the syphilitic saddle nose may be bridged up, and the "pug nose has received more attention since John O. Roe, of Rochester, explained its etiology and pathology, and suggested operative treatment." We cordially recommend this book to surgeons.

